# Ligand-based pharmacophore modeling and QSAR approach to identify potential dengue protease inhibitors

**DOI:** 10.3389/fmolb.2023.1106128

**Published:** 2023-02-23

**Authors:** Anushka A. Poola, Prithvi S. Prabhu, T. P. Krishna Murthy, Manikanta Murahari, Swati Krishna, Mahesh Samantaray, Amutha Ramaswamy

**Affiliations:** ^1^ Department of Biotechnology, M. S. Ramaiah Institute of Technology, Bengaluru, Karnataka, India; ^2^ Department of Pharmacy, Koneru Lakshmaiah Education Foundation, Vaddeswaram, Andhra Pradesh, India; ^3^ Department of Bioinformatics, Pondicherry University, Pondicherry, India

**Keywords:** Dengue, QSAR, pharmacophore modeling, docking, molecular dynamics

## Abstract

The viral disease dengue is transmitted by the Aedes mosquito and is commonly seen to occur in the tropical and subtropical regions of the world. It is a growing public health concern. To date, other than supportive treatments, there are no specific antiviral treatments to combat the infection. Therefore, finding potential compounds that have antiviral activity against the dengue virus is essential. The NS2B-NS3 dengue protease plays a vital role in the replication and viral assembly. If the functioning of this protease were to be obstructed then viral replication would be halted. As a result, this NS2B-NS3 proves to be a promising target in the process of anti-viral drug design. Through this study, we aim to provide suggestions for compounds that may serve as potent inhibitors of the dengue NS2B-NS3 protein. Here, a ligand-based pharmacophore model was generated and the ZINC database was screened through ZINCPharmer to identify molecules with similar features. 2D QSAR model was developed and validated using reported 4-Benzyloxy Phenyl Glycine derivatives and was utilized to predict the IC50 values of unknown compounds. Further, the study is extended to molecular docking to investigate interactions at the active pocket of the target protein. ZINC36596404 and ZINC22973642 showed a predicted pIC50 of 6.477 and 7.872, respectively. They also showed excellent binding with NS3 protease as is evident from their binding energy of −8.3and −8.1 kcal/mol, respectively. ADMET predictionsofcompounds have shown high drug-likeness. Finally, the molecular dynamic simulations integrated with MM-PBSA binding energy calculations confirmedboth identified ZINC compounds as potential hit moleculeswith good stability.

## Graphical Abstract

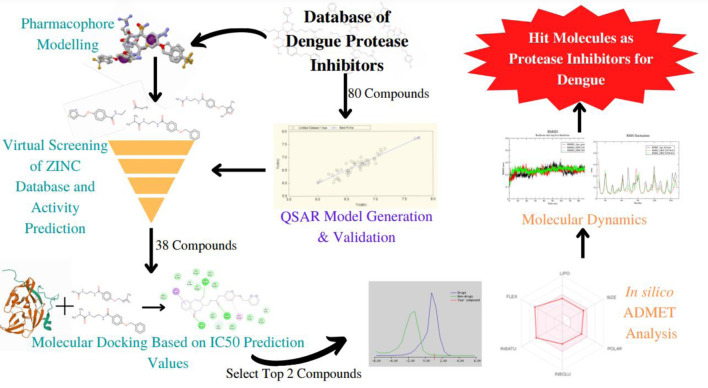

## Introduction

Dengue, a viral disease caused by members of the Flaviviridae family, is a leading public health concern, affecting most Asian and Latin American countries, and becoming a major cause of hospitalization and death in these regions (WHO, 2022). The disease spreads among humans through infected female *Aedes aegypti* or *Aedes albopictus *(Adawara et al., 2020). There are four serotypes of Dengue virus (DENV), namely, DEN-1, DEN-2, DEN-3, and DEN-4, of which DEN-2 is considered the most virulent strain (Adawara et al., 2020; Dwivedi et al., 2021). Up to date, other than supportive, no specific antiviral treatment exists to treat the illness, thus finding potential compounds that have an anti-dengue activity that can be developed into efficient drugs with the least toxic effects on human beings is the need of the hour (Wellekens et al., 2022). *In vitro* testing of inhibitory activities of various compounds is a time-consuming procedure and is also expensive, pointing toward the usage of quantitative structure-activity relationship (QSAR) models which is a promising way to predict the biological activity of new compounds (Kurniawan et al., 2020).

The viral genome encodes for three structural proteins and seven non-structural proteins, of which NS3 is a non-structural protein that is essential for RNA replication and viral assembly (Dwivedi et al., 2021). This protein contains a serine protease domain, whose activity depends on the formation of a non-covalent complex with the NS2B protein as a cofactor, thus making the NS3 protein an attractive target that can be used to develop dual-acting drugs that are effective against DENV (Behnam et al., 2015). It has been reported that structure-based drug design may not be suitable for developing NS3–NS2B inhibitors due to the specific structure of the protease which is slightly smooth in 3D space, and to date, ligand interaction mechanism and QSAR information are very limited (Luo et al., 2017).

Various *in silico* studies aiming to identify NS2B/NS3 inhibitors have been performed, for example, a study by Qamar et al., in 2017 pointed out that plant flavonoids have the potential to inhibit the dengue protease enzyme and could stop replication of DENV(Qamar et al., 2017). Other studies focusing on phytocompounds as novel dengue protease inhibitors have also been reported isolated phytochemicals belonging to different groups including fatty acids, glucosides, terpenes and terpenoids, flavonoids, phenolics, chalcones, acetamides, and peptides. Curcumin, quercetin, and myricetin were found to act as non-competitive inhibitors for the NS2b/NS3 protease enzyme (Saqallah et al., 2022). Though various *in silico* experiments have been performed to identify NS2b/NS3 inhibitors, most of these studies are molecular docking based, and studies based on QSAR are few.

In 2015, Behnam et al. performed a study that presents an extensive biological evaluation of NS3 inhibitors containing benzyl ethers of 4-hydroxyphenylglycine that function as non-natural peptide building blocks synthesized *via* a copper-complex intermediate. In this study, we make use of these inhibitors to develop a ligand-based pharmacophore model as well as a QSAR model, in order to identify lead compounds having anti-dengue activity. This study also elaborates on the ligand interactions and toxicity analysis of the inhibitors based on *in silico* predictions. These findings can then be utilized and integrated into *in vitro* studies in order to further confirm the possibility of developing these inhibitors into effective drugs.

## Methodology

### Identification of inhibitor compounds

An extensive survey of literature revealed the DenvInD-Database of inhibitors of Dengue virus (https://webs.iiitd.edu.in/raghava/denvind/), a curated database of Dengue virus inhibitors for clinical and molecular research (Dwivedi et al., 2021). This database contains detailed information about the SMILES, PubChem IDs, EC_50_, CC_50_, IC_50_, and K_i_ values of 484 compounds which have been validated as inhibitors against various drug targets of dengue virus using *in vitro* studies. From this database, the specific set of inhibitors against NS3 protease was selected for further studies. Out of the 365 NS3 protease inhibitors reported in the database, 104 compounds containing 4-Benzyloxy Phenyl Glycine residues were selected, whose biological assays were performed using fluorometric assay HPLC-based DENV-protease assay in order to eliminate false positives (Behnam et al., 2015). The IC_50_ value is a measure of the effectiveness of a drug in bringing about the inhibition of its respective target. Therefore, based on the availability of IC_50_ values, 80 compounds were further selected for the pharmacophore modeling and QSAR study as is presented in the supplementary information. The IC_50_ values were converted to pIC_50_ values in order to normalize the variation in concentration units. The structures of these 80 compounds were drawn using ChemSketch, a software developed by Advanced Chemistry Development, Inc. (Li et al., 2004).

### Identification of standard drugs

There is presently no standard treatment for dengue infection and therefore there is a need to explore all avenues that will lead us to potential drugs. In order to carry out a comparative analysis between the compounds obtained from DenvInD and standard drugs used to treat other similar viruses, as well as to check the possibility of drug repurposing, a set of 15FDA-approved standard antiviral drugs have been reported to inhibit protease in Hepatitis C Virus (HCV) and Human Immunodeficiency Virus (HIV) was identified, as shown in Table 1. The SDF files of these compounds were downloaded from DrugBank for further analysis (Wishart et al., 2018).

**TABLE 1:** Structures of the selected FDA approved drugs and their docking scores.

S. No.	Standard drug	Structure	Binding energy (kcal/mol)
1	Danoprevir	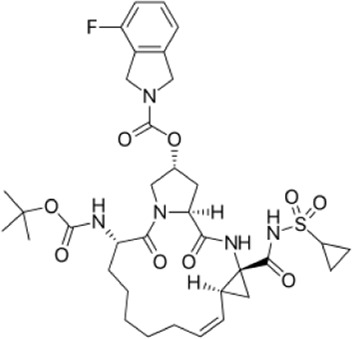	−13.5
2	Glecaprevir	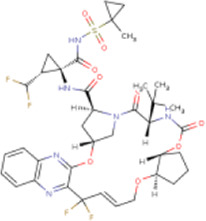	−13
3	Simeprevir	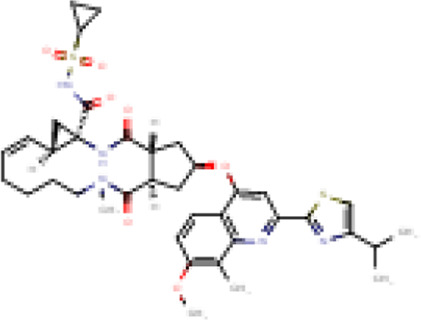	−12.1
4	Saquinavir	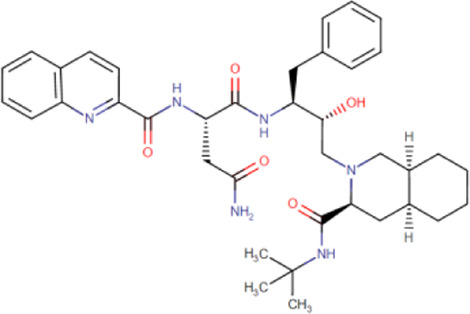	−10.5
5	Indinavir	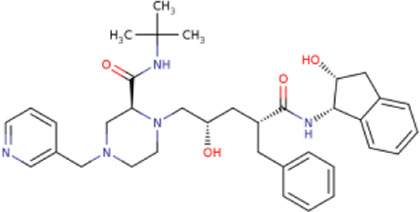	−10.5
6	Tipranavir	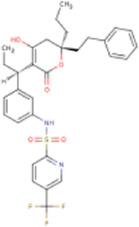	−10.3
7	Nelfinavir	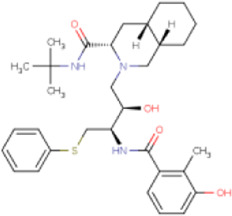	−10.2
8	Asunaprevir	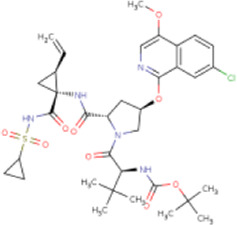	−9.9
9	Darunavir	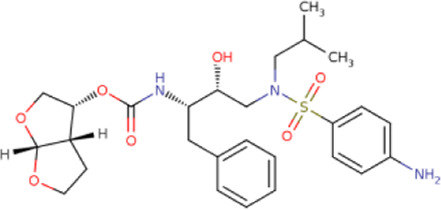	−9.4
10	Amprenavir	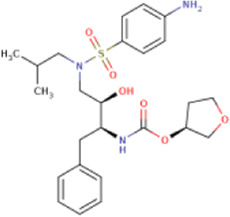	−9.3
11	Telaprevir	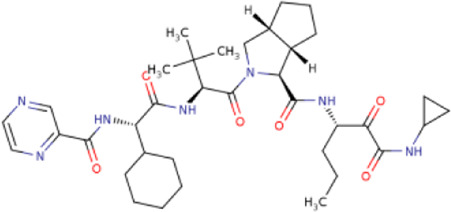	−9.2
12	Fosamprenavir	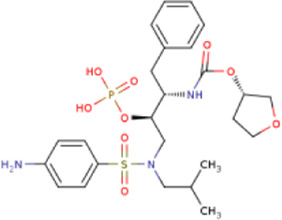	−9.2
13	Lopinavir	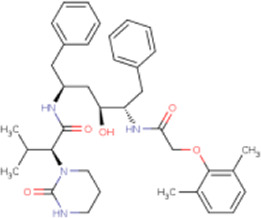	−9.1
14	Boceprevir	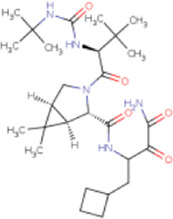	−8.8
15	Ritonavir	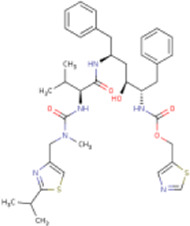	−8.6
16	Atazanavir	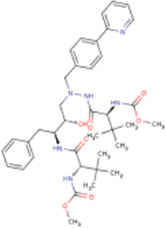	−8

### Pharmacophore-based screening of ZINC database

The top 3 compounds with the highest pIC50 values were selected and their energies were minimized using Avogadro, using the steepest descent algorithm and MMFF94 force field (Hanwell et al., 2012). These molecules were converted to mol2 format and were provided as input to PharmaGist with the maximum number of output pharmacophores as 5, in order to develop the pharmacophore model. The pharmacophore feature output file was then used as input to ZINCPharmer, an open web server used to screen the ZINC database to identify compounds with similar pharmacophore features (Koes and Camacho, 2012). The resultant compound hits were then downloaded as SDF files for molecular docking analysis.

### Quantitative structure-activity studies (QSAR) studies

#### Creating training and test set

The 80 final compounds chosen from DenvInD were split into training set and test set. The range of pIC50 values for the training set and test set was 5.42–7.74 and 5.01–7.55, respectively. Based on a randomized process, 64 compounds were considered in the training set, and the remaining 16 compounds were considered in the test set. The training set was used to build the QSAR model.

#### Generation of descriptor

Molecular descriptors refer to structural and physicochemical properties that define a molecule and usually include properties like steric parameters, hydrophobic properties, electrostatic properties, *etc.*, as well as constitutional properties of the molecule. The descriptors for the 64 compounds in the training set were calculated using PaDEL software (Yap, 2011). Significant descriptors were selected for further analysis based on their correlation with the pIC_50_ values of the training compounds.

#### Building QSAR model-generation and validation

The BuildQSAR tool was used to build the QSAR model using the 64 training compounds (Singh et al., 2022). A QSAR study performed First, a systematic search was performed to select a set of descriptors (maximum 3) on the basis of user-given correlation criteria with respect to activity (pIC_50_). Further, the Multiple Linear Regression (MLR) method was used to build the QSAR model using multiple combinations of the selected descriptors (Murahari et al., 2017). The descriptors were selected based on various statistical parameters like high correlation coefficient (R), high Fischer’s value (F-Test), low Standard error of estimate (s), statistical significance (p), high cross-validated square of correlation coefficient (Q^2^), low sum of squared error of prediction (SPRESS) and low standard deviation of error of prediction (SDEP). The models that showed significant statistical parameters were tested using the 16 compounds in the test set, to check the fitness of the QSAR model.

#### Activity prediction of screened ZINC compounds

The pIC_50_ values of ZINC database compounds obtained as a result of ZINCPharmerscreening were predicted using the validated QSAR model that showed highly significant statistical parameters. The compounds with good pIC_50_ values in comparison with compounds obtained from DenvInD were used for further computational studies.

### Molecular docking studies

#### Preparation of protein

The structure of Dengue Virus NS2B/NS3 Protease was obtained from RCSB PDB (PDB ID: 2FOM) (Sarwar et al., 2018). SWISS-MODEL was used to repair the missing atoms (Waterhouse et al., 2018). Further, the ligands from the protein structure were removed using BIOVIA Discovery Studio and the protein was prepared for docking in AutoDock Vina, a part of MGL tools 1.5.7 (Seeliger and De Groot, 2010; Pawar and Rohane, 2021). Water molecules were deleted, polar hydrogen atoms and Kollman charges were added. The prepared protein was saved as a pdbqt file and further used for docking analysis. The binding site coordinates were obtained as x = −3.243 y= −9.193 and z = 16.143 based on key amino acid residues (His 51, Asp 75, and Ser 135) using PyMol version 4.4, a molecular visualization software (Yuan et al., 2017). The grid box size of 40 A^0^ was used for docking.

#### Docking with ZINC database compounds and standard drugs

The compounds obtained from the ZINC database after the pharmacophore-based screening, as well as the 15FDA-approved antiviral protease inhibitors were converted to pdbqt format and their energy was minimized using the MMFF94 force field. AutoDock Vina was used for docking. Docking was performed using exhaustiveness parameter as 10. Docking scores and binding interactions at the active pocket of target protein for respective ligands were inspected and recorded carefully. The output complexes with high binding affinity and pIC_50_ were further used to perform molecular dynamics simulation studies.

### Molecular dynamic simulations

The top 2 compounds obtained after docking and QSAR activity predictions of the selected ZINC database compounds were further subjected to molecular dynamic simulations using GROMACS version 2018.1 (Van Der Spoel et al., 2005). The receptor topology was obtained by the “pdb2gmx” script, while the ligand topologies were obtained by the PRODRG server (Schüttelkopf and Van Aalten, 2004). Each of the generated ligand topologies was rejoined to the processed receptor structure to construct the ligand-protein complex. GROMOS96 54a7 force field was used to obtain the energy minimized conformations of all the processed complexes (Schmid et al., 2011). Next, a solvation step was performed wherein the structures were solvated in a cubic periodic box (90 Å, 90 Å, 90 Å) with water extended simple point charge (SPC) model. In order to neutralise the system, 4 Na ions added. Subsequently, energy minimization of the system was carried out for 50,000 steps using the steepest descent algorithm with <10.0 kJ/mol force. Upon energy minimization, equilibration of the system was performed with two consecutive steps. The NVT ensemble followed by NPT ensemble was done for 50,000 steps each. A constant temperature of 300 K and constant pressure of 1 atm were maintained through the entire MD simulation. The long-range electrostatic interactions were obtained by the particle mesh Eshwald method with a 12 Å cut-off and 12 Å Fourier spacing. Finally, the three well-equilibrated systems (one apo protein and two protein-ligand complexes) was subjected to a final 100 ns simulation. Root mean square deviation (RMSD), Root Mean Square Fluctuation (RMSF), Radius of Gyration (R g), Solvent Accessible Surface Area (SASA) and Number of Hydrogen bonds of the protein and complxes were calculated using gmx_rms, gmx_rmsf, gmx_gyrate, gmx_sasa and gmx_hbond tools, respectively. The MM/PBSA study using g_mmpbsa version 5.1.2 utility was used to analyze the binding free energy (ΔG binding) of the ligands with protein over the whole 100 ns simulation time.

### Prediction of drug-likeness and ADMET properties of ZINC compounds

The hit molecules were then studied further investigated for drug-likeness, toxicity, and ADME properties. Molsoft Drug-Likeness and molecular property prediction tool were used to predict drug-likeness (Elsherif et al., 2020) Other chemical properties like the number of hydrogen bond donors, hydrogen bond acceptors, BBB score, pK_a,_ etc., were also analyzed during this step. It is extremely important to understand the toxicity levels of compounds before considering it further as a potential drug lead. Hence to predict the toxicity class of compounds, ProTox-II was used (Drwal et al., 2014). Further, to elucidate the physicochemical descriptors, pharmacokinetic properties, ADME parameters, and drug-like nature, SwissADME tool was used (Daina et al., 2017).

## Results and discussion

### Ligand-based pharmacophore modeling

Top 3 compounds with highest pIC50, i.e., DenvInD_285, DenvInD_265 and DenvInD_266, were submitted to PharmaGistwebserver to generate the pharmacophore model. This web server predicts a ligand-based pharmacophore model based on the best alignment of maximum features between the submitted molecules. Considering a perfect alignment of all the 3 molecules submitted, a pharmacophore model was obtained with a PharmaGist score of 29.394 having six spatial features. The pharmacophore model generated includes a total of 6 features-spatial features, aromatic 2), donors 3), acceptor 1), and the results of other pharmacophores identified were presented in Table 2.

**TABLE 2:** PharmaGist results.

S. No.	Score	Spatial features	Aromatic	Hydrophobic	Donor	Acceptor	Molecules
1	29.394	6	2	0	3	1	DenvInD_285, DenvInD_266, DenvInD_265
2	22.780	6	1	1	3	1	DenvInD_285, DenvInD_266, DenvInD_265
3	22.045	4	2	0	1	1	DenvInD_285, DenvInD_266, DenvInD_265

### Pharmacophore-based screening of ZINC database

The pharmacophore features obtained from PharmaGist were downloaded and used to screen the ZINC database through ZINCPharmer webserver in order to find ligands with similar pharmacophore features with an assumption of having similarity in pharmacological properties. The query led to 38 hits from the ZINC database with optimization of low RMSD and molecular weight. The structures of these compounds were presented in the Supplementary Material.

### Building QSAR model and activity prediction of ZINC database compounds

Using PaDEL software 1,444 descriptors were generated for the training set of 64 compounds. Based on the correlation coefficient calculated with respect to pIC_50_ values of the respective compounds, 13 descriptors were identified for further analysis. The training set of 64 compounds was given as input to the BuildQSAR tool to generate the QSAR models. A variable selection search was performed using “systematic search” mode using correlation criteria limits of 0.6–0.78 and the variable limit of 3. The influencing parameters were found to be GATS6e (X1), GATS5i(X2), VE1_DzZ (X3), VE2_DzZ (X4), VE3_DzZ (X5), SpMAD_Dzp (X6), SpMax3_Bhp(X7), ETA_Epsilon_5 (X8), IC1(X9), IC2(X10), TIC0(X11), MIC1(X12), WTPT-3 (X13) and they are further described in Table 3. GATS6e and GATS5i are autocorrelation descriptors which are essentially molecular descriptors that encode molecular structure as well as the physicochemical properties attributed to the atoms in the form of vectors (Hollas, 2003). VE1_DzZ, VE2_DzZ, VE3_DzZ and SpMAD_Dzp are Barysz Matrix descriptors. Barysz matrix is a weighted distance matrix that accounts for the presence of multiple bonds and heteroatoms in the molecule under consideration. SpMax3_Bhp is a Burden Modified Eigenvalues descriptor that reflects the topology of the molecule. ETA_Epsilon_5 is an Extended Topochemical Atom descriptor that determines the contributions of specific positions within common substructures of molecular graphs towards total functionality (Roy and Ghosh, 2003). IC1, IC2, TIC0, and MIC1 are Information Content descriptors, and WTPT-3 is a PaDEL Weighted Path descriptor. The QSAR model was generated using a trial-and-error method to find the best fitting model that has a high R, R^2^, F-test, and Q^2^ and low s values, SPRESS, and SDEP statistical values. The top six models were shown in Table 4. These models were further tested using the test set to verify whether the pIC_50_ value predicted by these models was comparable to experimental values. Upon graphical analysis, it was seen that model 1 exhibited the highest R^2^ value of 0.703 between observed and predicted pIC_50_ values. Hence model 1 was chosen for further studies. The pIC_50_ predicted using Model 1 ranged from 4.507 to 8.164. Further information about the model is given in the supplementary file. The pIC_50_ of the library compounds ranged from 5.013 to 7.744. This shows that the validated QSAR model could identify compounds with better predicted pIC_50_ values, for which the objective was partially fulfilled. As the compounds need to be tested experimentally. The predicted activity for the ZINC database compounds were presented in Table 5. These compounds were then analyzed using docking studies to identify the binding patterns and interactions at the active pocket of the target protein.

**TABLE 3:** Details of the descriptors chosen to build the QSAR model (Karthikeyan et al., 2021).

S. No.	Descriptor	Description	Descriptor class
1	GATS6e	Geary autocorrelation–lag 6/weighted by Sanderson electronegativities	Autocorrelation descriptor
2	GATS5i	Geary autocorrelation–lag 5/weighted by first ionization potential	
3	VE1_DzZ	Coefficient sum of the last eigenvector from Barysz matrix/weighted by atomic number	Barysz Matrix descriptor
4	VE2_DzZ	Average coefficient sum of the last eigenvector from Barysz matrix/weighted by atomic number	
5	VE3_DzZ	Logarithmic coefficient sum of the last eigenvector from Barysz matrix/weighted by atomic number	
6	SpMAD_Dzp	Spectral mean absolute deviation from Barysz matrix/weighted by polarizabilities	
7	SpMax3_Bhp	Largest absolute eigenvalue of Burden modified matrix–n 3/weighted by relative polarizabilities	Burden Modified Eigen values descriptor
8	ETA_Epsilon_5	A measure of electronegative atom count	Extended Topochemical Atom descriptor
9	IC1	Information content index (neighborhood symmetry of 1-order)	Information Content descriptor
10	IC2	Information content index (neighborhood symmetry of 2-order)	
11	TIC0	Total information content index (neighborhood symmetry of 0-order)	
12	MIC1	Modified information content index (neighborhood symmetry of 1-order)	
13	WTPT-3	Sum of path lengths starting from heteroatoms	PaDEL Weighted Path descriptor

**TABLE 4:** QSAR models and their statistical parameters.

Model no.	Descriptor 1	Descriptor 2	Descriptor 3	R Value	R^2^ values	S	Q^2	F	*p*	SPRESS	SDEP	*n*	QSAR equation
1	X1	X7	X10	0.92	0.85	0.1537	0.8197	89.5853	0	0.1663	0.1614	53	Y1 = - 2.5236 (±0.4389) X1 - 0.0599 (±0.0592) X7 + 1.0876 (±0.3812) X10 + 4.5352 (±2.1729)
2	X1	X8	X10	0.92	0.85	0.1530	0.8215	90.5701	0	0.1654	0.1606	53	Y1 = - 2.5530 (±0.4378) X1 - 0.3144 (±0.2939) X8 + 1.0895 (±0.3795) X10 + 4.5728 (±2.1648)
3	X1	X5	X9	0.92	0.84	0.1521	0.8129	85.77733	0	0.1659	0.1610	52	Y1 = - 2.3301 (±0.4819) X1 + 0.0140 (±0.0127) X5 + 0.8225 (±0.4152) X9 + 6.5410 (±1.9935)
4	X1	X6	X10	0.91	0.83	0.1596	0.8053	82.5516	0	0.1718	0.1669	54	Y1 = - 2.5308 (±0.4532) X1 - 0.0097 (±0.0106) X6 + 1.0874 (±0.3936) X10 + 4.5341 (±2.2422)
5	X1	X5	X10	0.91	0.83	0.1607	0.8016	81.2388	0	0.1735	0.1685	54	Y1 = - 2.5155 (±0.4559) X1 + 0.0110 (±0.0136) X5 + 0.9397 (±0.4266) X10 + 5.1655 (±2.4390)

**TABLE 5:** Results of docking ZINC database compounds against NS3 protease.

S. No.	ZINC compound	Binding energy (kcal/mol)	Predicted pIC50
1	ZINC36596404	−9	6.477
2	ZINC22973642	−8.9	7.872
3	ZINC09789323	−8.7	6.399
4	ZINC16699623	−8.7	4.507
5	ZINC19143967	−8.5	7.047
6	ZINC09833225	−8.3	6.907
7	ZINC02458390	−8.2	6.189
8	ZINC06148003	−8.2	6.869
9	ZINC27672080	−8.2	7.086
10	ZINC14028064	−8.1	6.700
11	ZINC14037170	−8.1	7.188
12	ZINC35025967	−8	6.584
13	ZINC14036276	−8	6.860
14	ZINC67678868	−7.9	6.473
15	ZINC36656172	−7.9	7.474
16	ZINC02563681	−7.8	6.434
17	ZINC01155209	−7.8	6.743
18	ZINC15634648	−7.7	6.628
19	ZINC17795,206	−7.7	7.198
20	ZINC23080510	−7.7	8.050
21	ZINC32477936	−7.6	7.121
22	ZINC23327308	−7.6	7.414
23	ZINC32042479	−7.6	7.702
24	ZINC32908224	−7.6	7.391
25	ZINC14664807	−7.5	7.170
26	ZINC33242299	−7.5	7.713
27	ZINC69504947	−7.5	6.964
28	ZINC09826328	−7.5	6.728
29	ZINC23114768	−7.5	7.242
30	ZINC06445998	−7.4	5.955
31	ZINC37514943	−7.4	6.332
32	ZINC22755327	−7.4	7.666
33	ZINC32485749	−7.4	7.466
34	ZINC78464608	−7.4	8.164
35	ZINC32908634	−7.3	7.931
36	ZINC64718088	−7.3	6.723
37	ZINC23114770	−7.3	7.242
38	ZINC93765844	−7.3	6.838

### Molecular docking studies

#### Docking of ZINC database compounds

**FIGURE 1:**
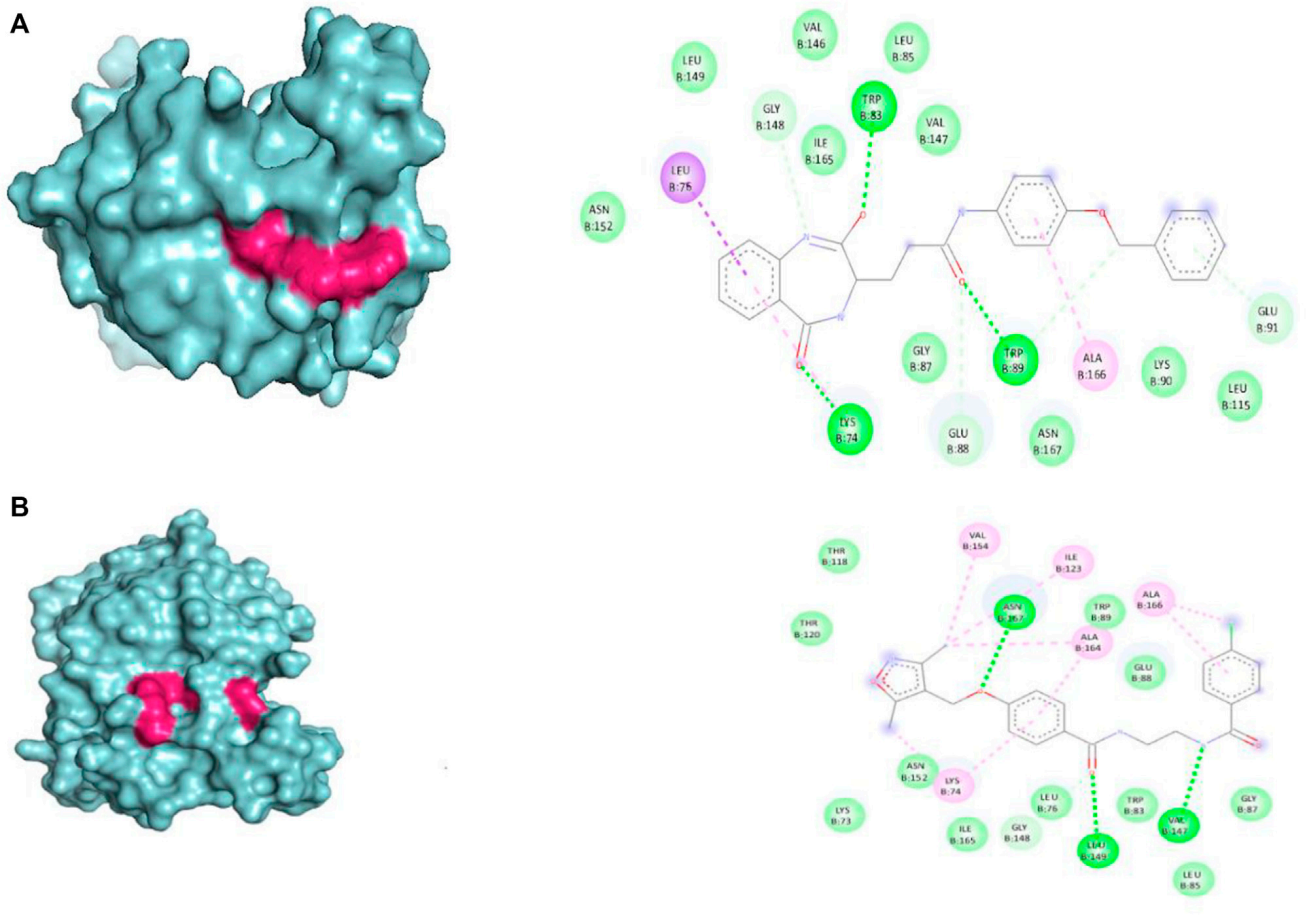
Visual representation of the docked complexes and the amino acid interactions of **(A)** ZINC36596404 **(B)** ZINC22973642.

The selected set of ZINC database compounds was subjected to docking against dengue protease as stated in the protocol. The binding energies ranged from −9 kcal/mol to −7.3 kcal/mol as shown in Table 5. The top 2 compounds identified were ZINC36596404 and ZINC22973642 with binding energies −9 kcal/mol and −8.9 kcal/mol, respectively. The interactions between the protein and the ligand were summarized in Table 6. Upon observing the interaction between dengue protease and ZINC36596404, conventional hydrogen bond, carbon-hydrogen bond, Pi-donor hydrogen bond, pi-sigma, and pi-alkyl were found to be significant. Lys74, Trp83 and Trp89 were involved in a conventional hydrogen bond, Gly148, Glu88 and Glu91 were involved in carbon-hydrogen bond and pi-donor hydrogen bond, Leu76 was involved in pi-sigma bond and Ala166 in pi-alkyl bond. Next, the interaction between dengue protease and ZINC22973642 was analyzed, revealing that van der Waals, conventional hydrogen bond, carbon hydrogen bond, alkyl, and pi-alkyl were noteworthy. The amino acid interactions for these bonds were seen to involve Thr118. Thr120, Trp89, Glu88, Asn152, Lys73, Ile165 for van der Waals bonds; Asn167, Leu149, Val47 contributed to conventional hydrogen bonding; Gly148, Leu76, Trp83, Gly87, Leu85 for hydrogen bonds; Val154, Ile123, Ala166, Ala164, Lys74 for alkyl and pi-alkyl. The interactions are represented in Figure 1.

**TABLE 6:** Summary of protein-ligand interactions.

S. No.	Compound	Residues involved in protein-ligand interactions								
		Conventional hydrogen bond	Carbon hydrogen bond	Pi-donor hydrogen bond		Pi-sigma	Pi-alkyl	Alkyl bonds	Van der waals	Hydrogen bond
1	ZINC36596404	Lys74, Trp83 and Trp 89	Gly148 and Glu88 and Glu91			Leu76	Ala166	-	-	-
2	ZINC22973642	Asn167,Leu149,Val47	-		-	-	Val154, Ile123, Ala166, Ala164, Lys74		Thr118, Thr120, Trp89, Glu88, Asn152, Lys73, Ile165	Gly148, Leu76, Trp 83, Gly87, Leu85
3	ZINC09789323	ASN152	-		-	-	Lys90, Ala166, Leu76		Leu115, Glu91, Trp89, Lys74, Ala164, Leu149, gly148, Met49	-

#### Docking of standard drugs

The results obtained when the 15 chosen standard drugs were docked against the Dengue protease were presented in Table 1. The binding energies fall in the range of −13.5 kcal/mol to −8 kcal/mol. From this, we can observe that Danoprevir, Glecaprevir, Simeprevir, Indinavir, Tipranavir, Nelfinavir, Asunaprevir, Darunavir, and Amprenavir have a better binding affinity with the Dengue protease compared to the ZINC database compounds screened in this study. This directs us to conduct an experimental study in order to formulate a drug that works against dengue protease. Danoprevir interacts with the receptor using van derWaals forces contributed by Asn167, Ala166, Ala164, Ile165, Asn152, Leu76, Met49, Leu149, Gly148 and Val147. Conventional hydrogen bonds made by Lys74 and carbon hydrogen bonds made by Leu85, Val146 and Gly87 also take part in the interactions. Glecaprevir interacted with the receptor through attractive charges of Glu88, conventional hydrogen bond of Trp83, carbon hydrogen bond of Gly148, halogen bond by Val147 and pi-cation bond by Glu88. Amino acids in Simeprevir that interacted with the receptor include Lys74, Asn167, Lys73, Ala164, Asn152, Ile123, Gly153, Val154, Thr120, Thr118, Asn119 and Val155 that contribute to van der waals forces, and Asp71 that is involved in attractive charges. Indinavir was seen to interact with the receptor through mainly alkyl and pi-alkyl bonds formed by Trp83, Leu149, Leu76 and Leu85, attractive charges of Glu88, and carbon hydrogen bond formed by Gly148 and Ala164. The amino acid interactions seen among other standard drugs studies are elaborated in the Supplementary Information. The binding interactions of Danoprevir and Glecaprevir, the top 2 compounds were further examined and compared with the binding interactions of the top hit ZINC compounds ZINC36596404 and ZINC22973642. Comparing the amino acid interaction of ZINC compounds and standard drugs with the receptor, we get interesting inferences. The results show that Ala166, Leu76, and Gly148 seem to play an important role in interaction with the receptor as they are involved in interactions with the receptor in Danoprevir, ZINC36596404, and ZINC22973642. While Ala166 is involved in van der Waals forces in Danoprevir interaction, it is involved in pi-alkyl and alkyl bonding in ZINC36596404 and ZINC22973642 interactions, but we can conclude that they are important residues in hydrophobic interactions. Leu76 and Gly148 seem to be contributing significantly to different types of hydrogen bonding. Glu88 and Trp83 were identified as another set of important amino acid residues interacting with the receptor in Glecaprevir, ZINC36596404, and ZINC22973642. Glu88 can be said to be necessary for hydrophobic interactions like pi-cation interaction and van der Waals interactions as well as hydrogen bonding. Trp83 has shown to be contributing to various hydrogen bonds in Glecaprevir, ZINC36596404, and ZINC22973642. Gly148 can be pointed out as a major key residue as it is involved in hydrogen bonding in all the compounds discussed above. From this, we can understand that by preserving these key interactions in the ZINC compounds and modifying other groups, we can develop the identified ZINC compounds into effective inhibitors of Dengue Protease.

### Molecular dynamic simulation

#### Root mean square deviation analysis

**FIGURE 2:**
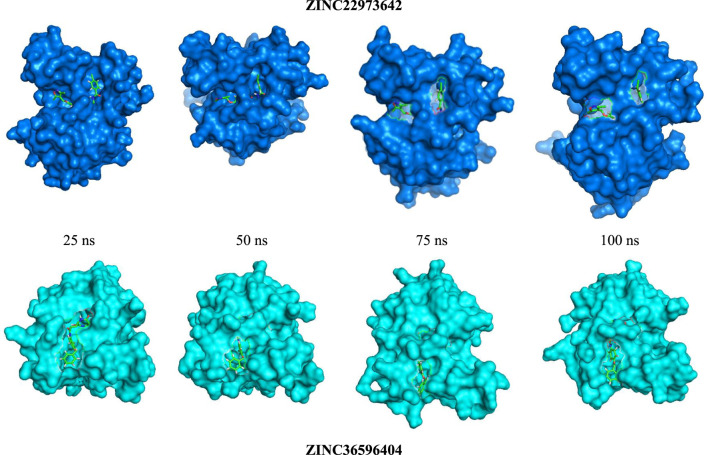
RMSD study of top 2 ligands for 100 ns MD Simulation.

ZINC36596404 and ZINC22973642 with the lowest binding energies were subjected to molecular dynamics simulation in order to analyze the flexibility and stability of the protein-ligand complexes in a cellular atmosphere. The changes in the complex structure and conformation were assessed for a simulation time frame of 100 ns through MD simulations. Differentparameters like RMSD, RMSF, R_g_, SASA were determined to understand the stability of the molecular trajectory, flexibility, ligand-receptor affinity and the extent of compactness and folding behavior. Figure 2 shows the pose of respective ligand during MD simulations in the active pocket at 25, 50, 75 and 100 ns, respectively. Supplementary Figure S3 summarizes the results obtained. RMSD evaluates whether the complex system has equilibrated and attained stability over the time duration of the simulation. In the case of apo-protein, the RMSD values showed a general increasing trend from 0 to 1.6 ns with RMSD values from 0 to 0.194 nm. Thereafter, the values showed slight variations of small magnitude. Towards the end of the simulation, particularly after 50 ns, a fairly constant value that remained between 0.2 and 0.24 nm was obtained. Considering the ZINC22973642 compound, the RMSD values showed a general increasing trend till 19.68 ns, with RMSD values ranging from 0 to 0.27 nm. From this point ahead, the values remained fairly constant in the range between 0.2 and 0.24 nm. The compound ZINC36596404 showed relatively better stability, as the results show an increase followed by decreasing trend until around 30 ns and thereafter remains at an almost constant value of 0.23 nm with only slight variations.

#### Root mean square fluctuation analysis

RMSF values for C_α_ atoms were calculated and comparatively analyzed for the ligand-bound complexes along with that of the apo-protein in order to look into the mean residual fluctuations, motion, and flexibility of the amino acid residues of particular regions of the ligand binding during the simulation time. Supplementary Figure S4 shows the results obtained. It was observed that about seven amino acids (Gly62, Val72, Lys104, Gly114, Gly121, Pro132, Gly153) are directly involving in the complex formation *via* interactions like conventional hydrogen bonds, carbon hydrogen bonds, Pi-donor hydrogen bond,Pi-sigma,Pi-alkyl, Van der Waals, *etc.* From the figure we can see that these residues are decreased in the complex due to the ligand binding properties when compared to their free dynamics in the apoprotein. From this, it is understood that the apo-protein, ZINC22973642, and ZINC36596404 show a very similar pattern where maximum residues show fluctuations, however, the vacillation was less than 0.3 nm for a majority of these residues.

#### Radius of gyration (R_g_)

The radius of gyration refers to the root mean square distance of the atoms from their rotational axis. It helps to gatherdetails about the compactness, rigidity, and folding behavior of the receptor during the time frame of the simulation. Lower R_g_ valuesshow that minimal fluctuations indicate a stable protein-ligand complex. Higher R_g_ values along with variation suggests instability of the complex. The values of R_g_ obtained are pictorially represented in Supplementary Figure S5. ZINC22973642 and ZINC36596404 happen to show a similar R_g_ pattern where the value remains fairly constant at 1.65 nm with very minor variations. From these results, we can conclude that the protein attained a compact state and does not show abrupt fluctuations indicating that a stably folded protein is formed upon binding of ligands to the ZINC database compounds.

#### Solvent accessible surface area

The binding of small molecules to receptor protein induces certain structural and conformational changes which have an impact on the protein volume. This change can indirectly give an insight into the protein-ligand complex during the simulation. SASA was calculated to look into the solvent behavior of the dengue protease upon binding to the ligands and it was comparatively analyzed to the changes in surface area of the apo-protein. Hydrophobic residues contribute to SASA values. The exposure of these residues from their hydrophobic core region leads to complex instability by decompressing the receptor. Similar to R_g_, lower and minimal fluctuations in the values indicated stabilized, compressed and correctly folded target protein. The SASA values were calculated and plotted against time in Supplementary Figure S6. The apo-protein exhibited minimal fluctuations in SASA values until around 50,000 ps from where it started increasing up until 60,000 ps and further decreased until the values stabilized. Both the ZINC database compounds showed a closely similar pattern of minimal fluctuations in the SASA values throughout the simulation period.

#### Hydrogen bonds

The binding affinity of identified small molecules with the target protein can be ascertained by hydrogen bond formation. The number of hydrogen bonds formed between ligand and dengue protease revealed the binding affinity. Graphical results were presented in Supplementary Figure S7. ZINC22973642 showed an average binding affinity with the protein and formed a maximum of 7 hydrogen bonds throughout the simulation period. ZINC36596404 had higher binding energy with the protein and this is clearly explained by the consistent hydrogen bond formation with the protein. From the figure, we can see that the ZINC compounds consistently maintain at least 5 hydrogen bonds throughout the simulation period. The residues involved in hydrogen bonding in ZINC36596404 were Lys74, Trp83 and Trp89 which were involved in a conventional hydrogen bond, Gly148, Glu88 and Glu91 which were involved in carbon-hydrogen bond and pi-donor hydrogen bond. Similarly, for ZINC22973642, Asn167, Leu149, Val47 contributed to conventional hydrogen bonding, and, Gly148, Leu76, Trp83, Gly87, Leu85 for hydrogen bonds. The complexes eventually stabilized, as it can be interpreted from the structural parameters.

#### MM-PBSA binding free energy

One of the widely accepted methods for estimation of binding free energy of small ligands with biological macromolecules is Molecular Mechanics Poisson Boltzmann Surface Area continuum solvation (MM-PBSA). The energy values obtained were summarized in Table 7. For both the ZINC database compounds, SASA energy contributed more significantly towards the binding as compared to Electrostatic energy and van der Waal energy. In both cases, polar solvation energy seems to be positively influencing the binding and hence we can say that it does not favorably benefit the binding. In conclusion, the results of the molecular dynamics simulation show that both ZINC36596404 and ZINC22973642 have a good affinity and binding stability towards the targeted dengue protease.

**TABLE 7:** MM-PBSA values of the two complexes after 100 ns simulation.

S. No.	Energy terms (KJ/mol)	ZINC22973642	ZINC36596404
1	Van der Waal	−241.848 ± 0.791	−250.309 ± 1.106
2	Electrostatic	−87.760 ± 1.045	−104.692 ± 1.163
3	Polar solvation	220.611 ± 16.207	261.191 ± 22.538
4	SASA	−23.200 ± 0.055	−23.788 ± 0.072
5	Binding energy	−132.196 ± 16.764	−116.651 ± 21.635

### Prediction of drug likeliness and ADMET properties

The drug-likeness of ZINC36596404 predicted using Molsoft showed a score of 0.43. From the results, 5 hydrogen bond acceptors and 3 hydrogen bond donors were also identified. The BBB score was reported as 2.22 which is on the lower side. The drug-likeness of ZINC22973642 analyzed by Molsoft had a score of 1.10. This drug-likeness score is predicted Molsoft’s chemical fingerprints made using a dataset containing 5,000 marketed drugs and 10,000 non-drug compounds. The drug-likeness value ranges from −1 to +1, where values equal to or less than 0 indicates that the compound does not seem to be a likely drug, whereas values greater than 0 indicate good drug-likeness of the compound. Since both the compounds discussed here have positive drug-likeness scores, we can say that they seem to be drug-like. The results also identified 5 hydrogen bond acceptors and 2 hydrogen bond donors. The BBB score was 2.85 and is on the lower side, similar to the previous compound. ZINC36596404 belongs to toxicity class 5 indicating that it may be harmful if swallowed (2000 < LD50 ≤ 5,000) and ZINC22973642 to class 4 signifying that it may be harmful if swallowed (300 < LD50 ≤ 2000) as per predictions made by ProtoxII. The ADME results obtained from SwissADME are shown in Table 8. ZINC22973642 shows no violation of Lipinski’s rule of five. It is seen to have good GI absorption, good solubility, and low BBB permeability indicating that it does not cross the blood-brain barrier. It is seen to inhibit CYP1A2, CYP2C19, CYP2C9, CYP2D6, and CYP3A4 which are cytochrome enzymes involved in the detoxification and metabolism of drugs. The skin permeation parameter for this compound indicates that it is moderately good for topical applications. Its bioavailability score shows that it is sufficiently absorbable and available throughout the body when administered *via* the oral route. The predicted LD50 is also sufficiently high. This, coupled with a good drug-likeness score, makes this compound a very potent lead that can be further explored and developed into an efficient drug against dengue protease. ZINC36596404 also shows similar properties as that of ZINC22973642, but only differs in that it does not inhibit CYP1A2. The fact that these two ZINC compounds showed good binding stability and affinity to Dengue Protease, combined with their positive drug-likeness, show that these compounds can be studied further *in vitro* in order to develop them into effective anti-Dengue drugs.

**TABLE 8:** Drug-likeness and ADMET properties of top 2 compounds.

S.No.	Parameter	ZINC22973642	ZINC36596404
1	Number of Hydrogen Bond Acceptors	5	5
2	Number of Hydrogen Bond Donors	2	3
3	BBB Score	2.85	2.22
4	Drug-likeness model score	1.1	0.43
5	Solubility	3.44e-05	4.09e-05
6	GI absorption	High	High
7	CYP1A2 inhibitor	Yes	No
8	CYP2C19 inhibitor	Yes	Yes
9	CYP2C9 inhibitor	Yes	Yes
10	CYP2D6 inhibitor	Yes	Yes
11	CYP3A4 inhibitor	Yes	Yes
12	Log Kp (skin permeation)	−6.42 cm/s	−6.67 cm/s
13	Bioavailability score	0.55	0.55
14	LD50	586 mg/Kg	3000 mg/kg
15	Toxicity class	4	5

## Conclusion

In this study, a ligand-based QSAR and pharmacophore model of Dengue protease inhibitors was developed using 4-Benzyloxy Phenyl Glycine derivatives. The GATS6e, GATS5i, VE1_DzZ, VE2_DzZ, VE3_DzZ, SpMAD_Dzp, SpMax3_Bhp, ETA_Epsilon_5, IC1, IC2, TIC0, MIC1, WTPT-3 descriptors were seen to have an effect on the anti-dengue protease activity. The validated QSAR model showed significant statistical parameters and can be used to predict the activity of unknown compounds for anti-dengue protease activity. Using this QSAR model and the pharmacophore features presented above, other 4-Benzyloxy Phenyl Glycine derivatives can be modified to enhance their activities. This model can be a helpful tool to reduce the time and expense involved in dengue protease antagonist synthesis and activity determination. Further, the molecular docking and dynamics simulation studies performed using the compounds identified from the ZINC database have indicated that ZINC36596404 and ZINC22973642 show excellent binding with the dengue protease. The complexes also show structural stability. They also have good drug-likeness and compatible ADMET properties. It can be inferred that these two compounds form promising candidates in the development of dengue protease antagonists. Further work that aims to test the *in vitro* and *in vivo* effects of these two compounds is required in order to validate these results. Thus, our findings, coupled with laboratory testing of the identified potential leads can help to develop strong antagonists for dengue protease.

## Data Availability

The datasets presented in this study can be found in online repositories. The names of the repository/repositories and accession number(s) can be found in the article/Supplementary Material.
