# Supplementary material for: Ligand-based pharmacophore modeling and QSAR approach to identify potential dengue protease inhibitors
Source: Front Mol Biosci. 2023 Feb 23;10:1106128. doi: 10.3389/fmolb.2023.1106128 (PMC9996041; doi:10.3389/fmolb.2023.1106128)
Supplement: Supplementary file 1 [file DataSheet1.PDF]

Supplementary Information:

Table of structures and pIC_{50} values of the NS3 inhibitors used to build the QSAR models (Behnam et al., 2015)

**Test Data Set**			
**S. No.**	**DenvInD_ID**	**Structure**	**pIC_{50}**
1.	DenvInD_163	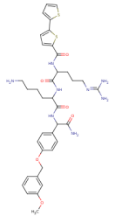	6.754487332
2.	DenvInD_217	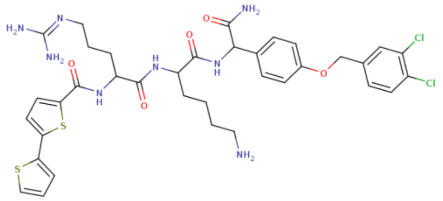	6.66756154
3.	DenvInD_218	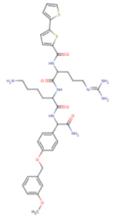	6.754487332
4.	DenvInD_227	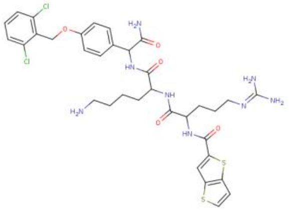	6.801342913

5.	DenvInD_228	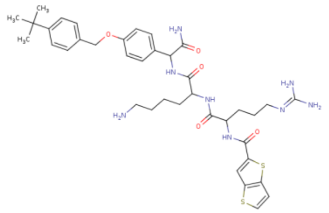	6.804100348
6.	DenvInD_230	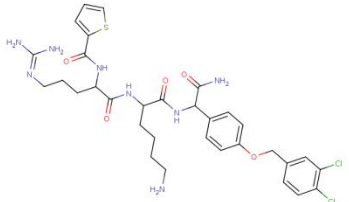	7.102372909
7.	DenvInD_231	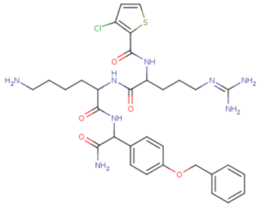	6.71219827
8.	DenvInD_233	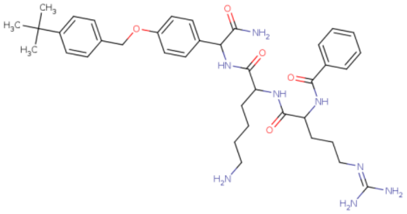	6.761953897
9.	DenvInD_234	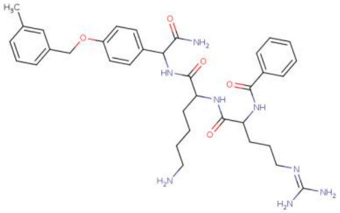	6.510041521

10.	DenvInD_235	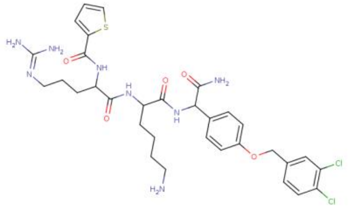	7.102372909
11.	DenvInD_236	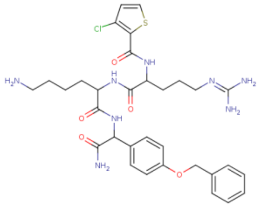	6.71219827
12.	DenvInD_237	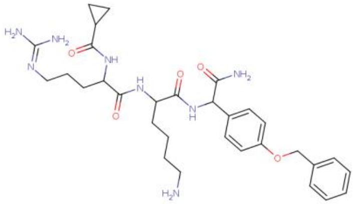	6.09420412
13.	DenvInD_238	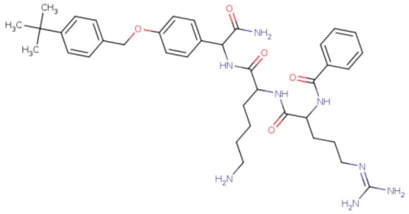	6.761953897
14.	DenvInD_239	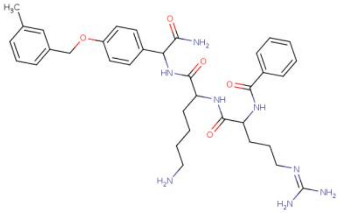	6.510041521

15.	DenvInD_240	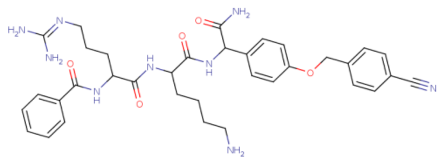	6.399027104
16.	DenvInD_241	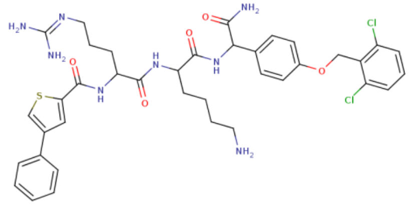	6.88941029
17.	DenvInD_242	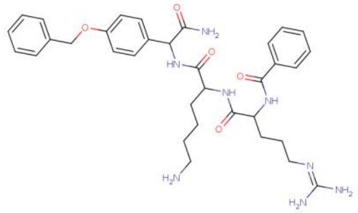	6.029188389
18.	DenvInD_244	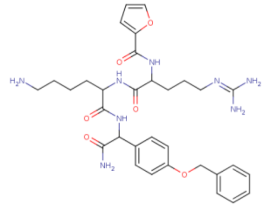	6.319664487
19.	DenvInD_245	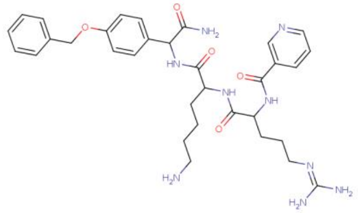	6.581698709

20.	DenvInD_246	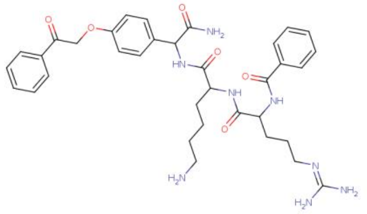	6.313363731
21.	DenvInD_247	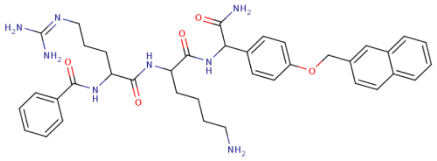	6.716698771
22.	DenvInD_248	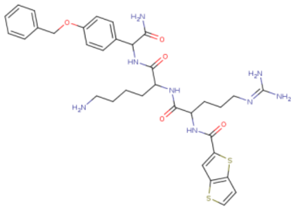	6.396855627
23.	DenvInD_251	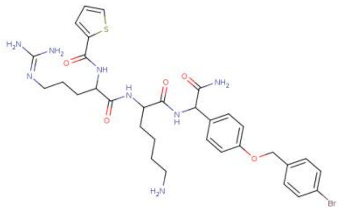	7.148741651
24.	DenvInD_252	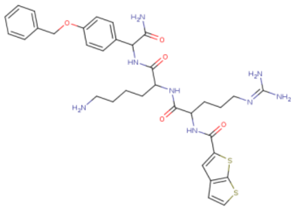	6.296708622

25.	DenvInD_253	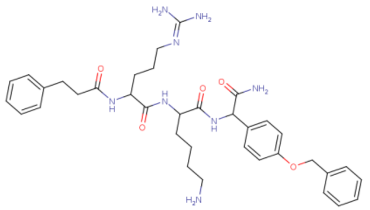	6.042871802
26.	DenvInD_254	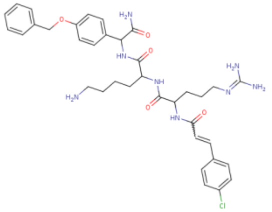	6.142064735
27.	DenvInD_255	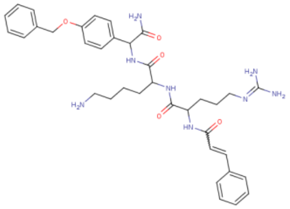	6.490797478
28.	DenvInD_256	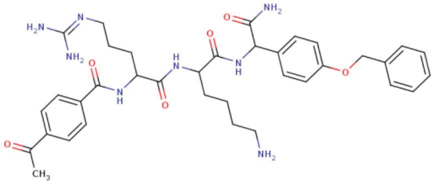	5.983384452
29.	DenvInD_257	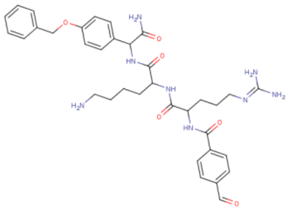	6.634512015

30.	DenvInD_259	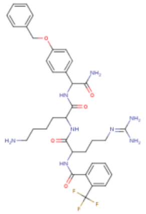	6.521433504
31.	DenvInD_261	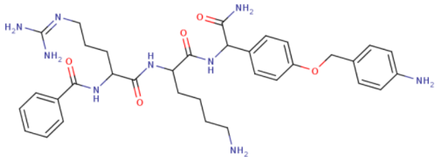	5.700492701
32.	DenvInD_262	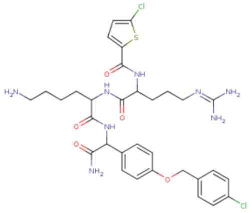	6.954677021
33.	DenvInD_263	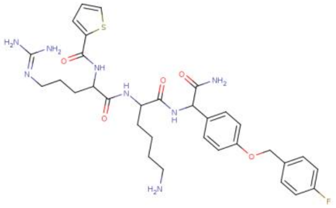	7.214670165
34.	DenvInD_264	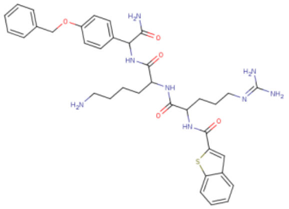	6.345823458

35.	DenvInD_265	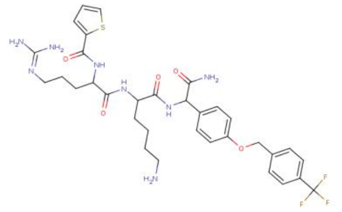	7.721246399
36.	DenvInD_267	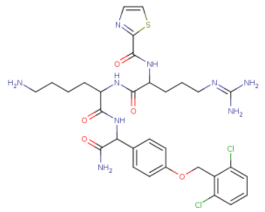	7.301029996
37.	DenvInD_268	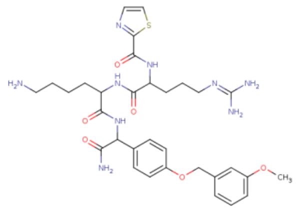	7.327902142
38.	DenvInD_269	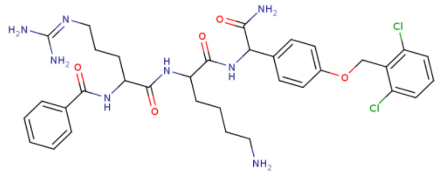	6.821023053
39.	DenvInD_271	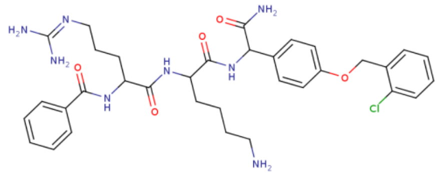	6.493494968

40.	DenvInD_272	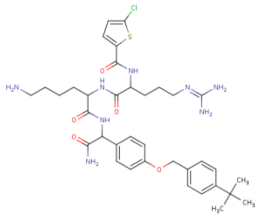	6.886056648
41.	DenvInD_273	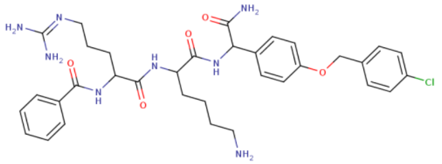	6.844663963
42.	DenvInD_274	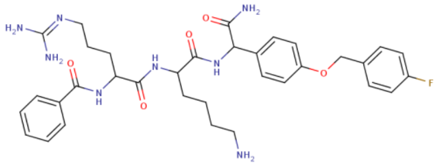	6.707743929
43.	DenvInD_275	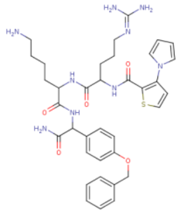	6.492144128
44.	DenvInD_276	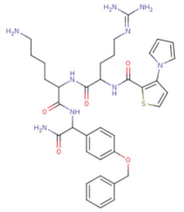	6.425968732

45.	DenvInD_277	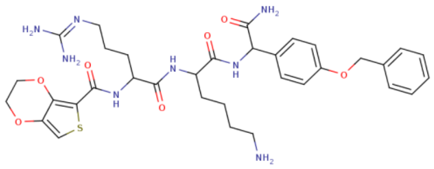	6.71219827
46.	DenvInD_278	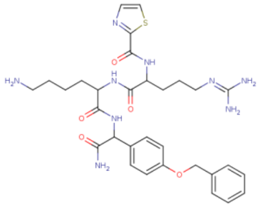	7.004364805
47.	DenvInD_279	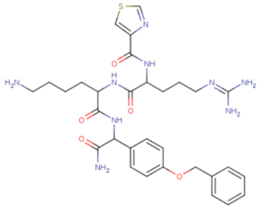	6.761953897
48.	DenvInD_280	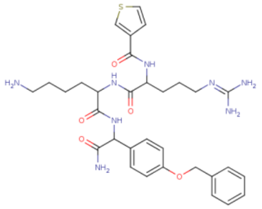	6.747146969
49.	DenvInD_281	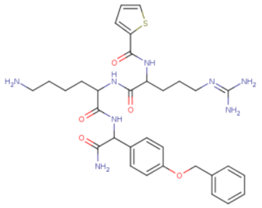	6.962573502

50.	DenvInD_282	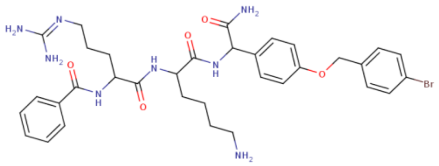	6.627087997
51.	DenvInD_283	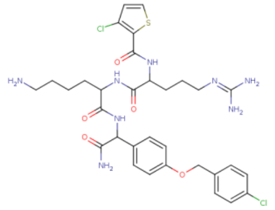	6.920818754
52.	DenvInD_285	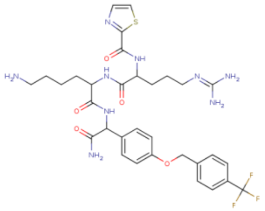	7.744727495
53.	DenvInD_286	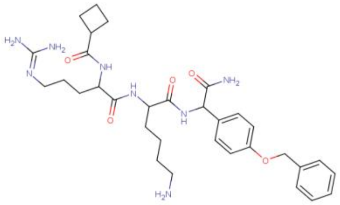	6.199970641
54.	DenvInD_287	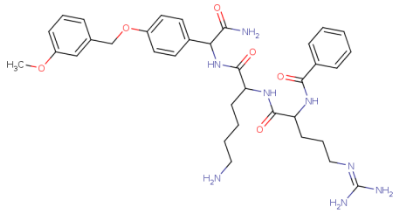	6.832682665

55.	DenvInD_288	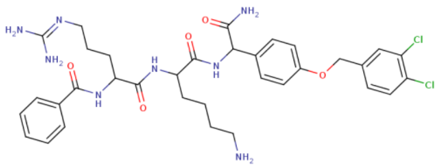	6.752026734
56.	DenvInD_289	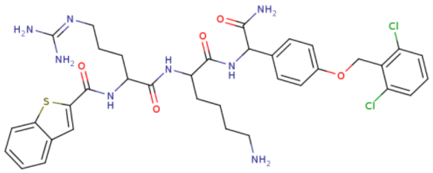	6.73754891
57.	DenvInD_291	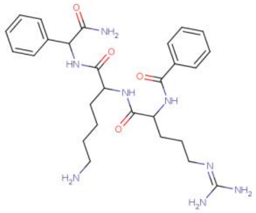	5.42136079
58.	DenvInD_292	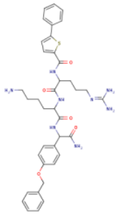	6.459670525
59.	DenvInD_293	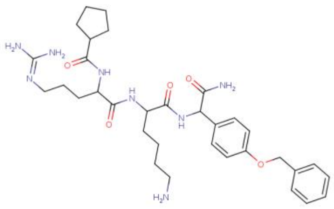	6.154281982

60.	DenvInD_294	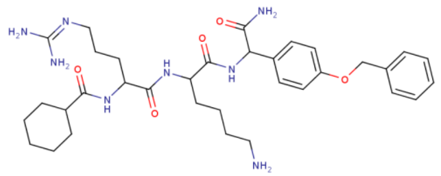	6.107348966
61.	DenvInD_295	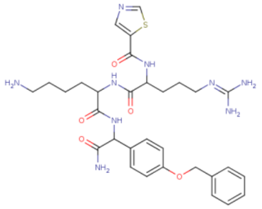	6.517126416
62.	DenvInD_296	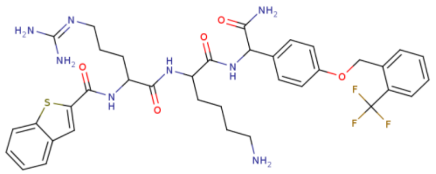	6.614393726
63.	DenvInD_297	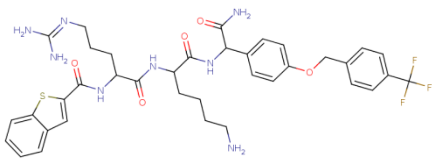	6.701146924
64.	DenvInD_300	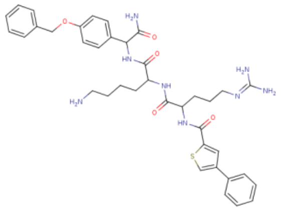	6.517126416

Validation Data Set			
**S. No.**	**DenvInD_ID**	**Structure**	**pIC$_{50}$**
1.	DenvInD_226	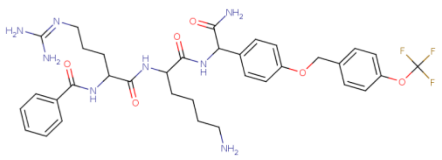	6.739928612
2.	DenvInD_243	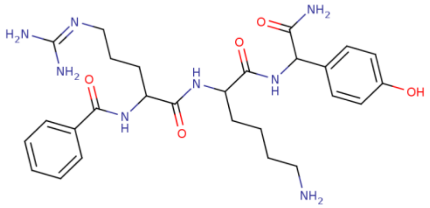	5.692076296
3.	DenvInD_219	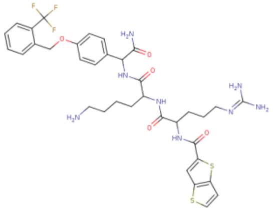	6.705533774
4.	DenvInD_258	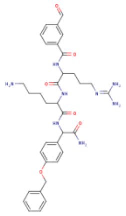	6.785156152

5.	DenvInD_298	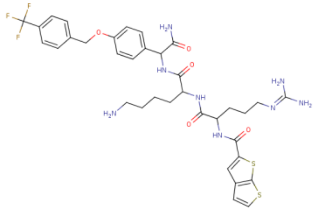	6.692503962
6.	DenvInD_301	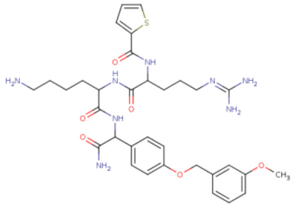	7.167491087
7.	DenvInD_250	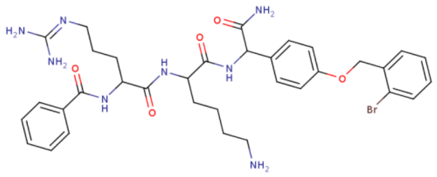	6.436518915
8.	DenvInD_232	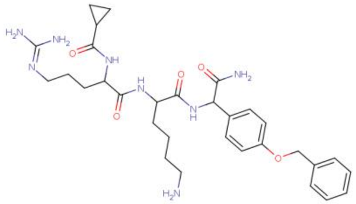	6.09420412
9.	DenvInD_249	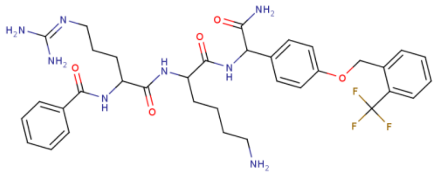	6.742321425

10.	DenvInD_266	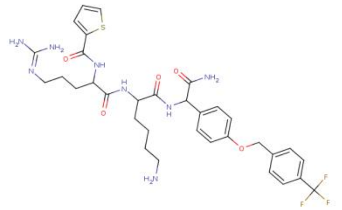	7.552841969
11.	DenvInD_290	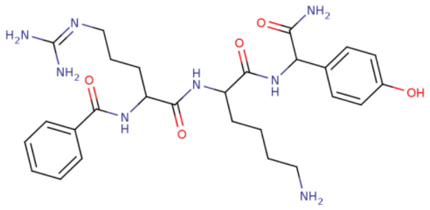	5.013452187
12.	DenvInD_299	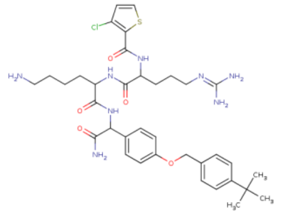	6.91721463
13.	DenvInD_260	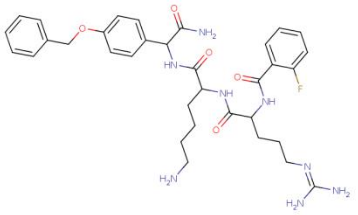	6.468521083
14.	DenvInD_229	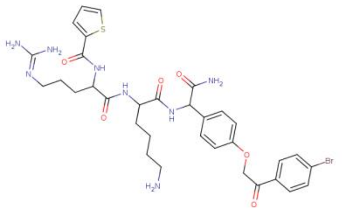	6.829738285

15.	DenvInD_225	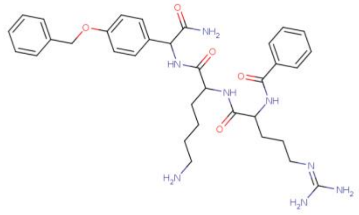	6.435333936
16.	DenvInD_270	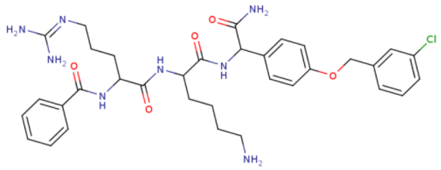	6.730487056

Table of structures of the 38 ZINC compounds obtained after pharmacophore screening

**S. No.**	**Compound**	**Structure**
1.	ZINC78464608	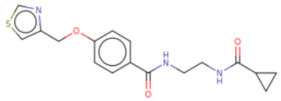
2.	ZINC23080510	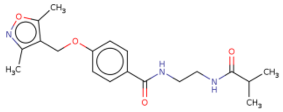

3.	ZINC32908634	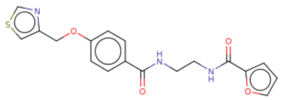
4.	ZINC22973642	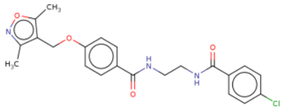
5.	ZINC33242299	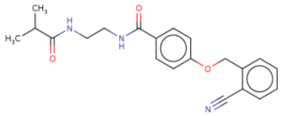
6.	ZINC32042479	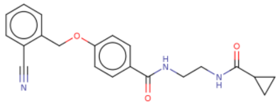
7.	ZINC22755327	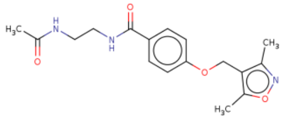
8.	ZINC36656172	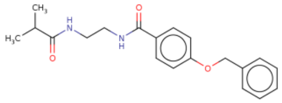
9.	ZINC32485749	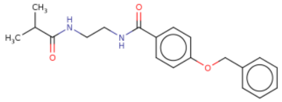

10.	ZINC23327308	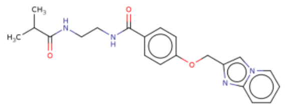
11.	ZINC32908224	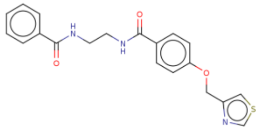
12.	ZINC23114770	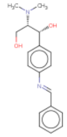
13.	ZINC23114768	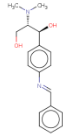
14.	ZINC17795206	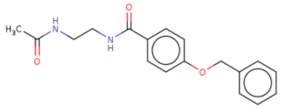
15.	ZINC14037170	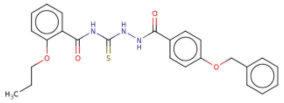
16.	ZINC14664807	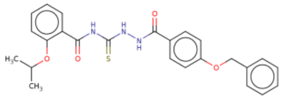

17.	ZINC32477936	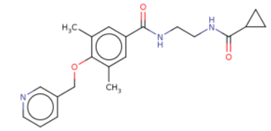
18.	ZINC27672080	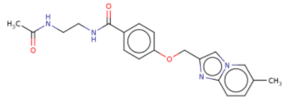
19.	ZINC19143967	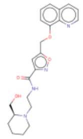
20.	ZINC69504947	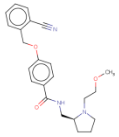
21.	ZINC09833225	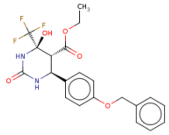
22.	ZINC06148003	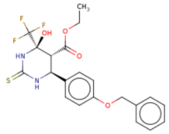
23.	ZINC14036276	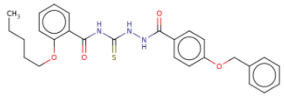

24.	ZINC93765844	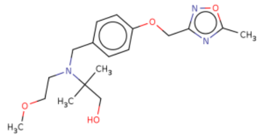
25.	ZINC01155209	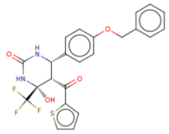
26.	ZINC09826328	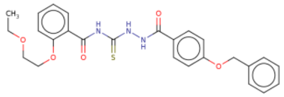
27.	ZINC64718088	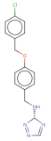
28.	ZINC14028064	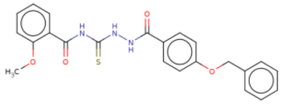
29.	ZINC15634648	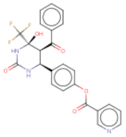
30.	ZINC35025967	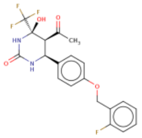

31.	ZINC36596404	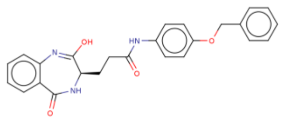
32.	ZINC67678868	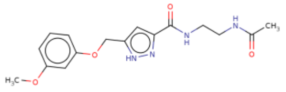
33.	ZINC02563681	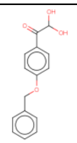
34.	ZINC09789323	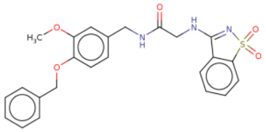
35.	ZINC37514943	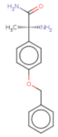
36.	ZINC02458390	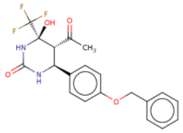
37.	ZINC06445998	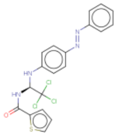

38.	ZINC16699623	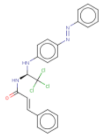

Predictions based on QSAR model 1 and graphical results

Equation: Y1 = - 2.5236 (± 0.4389) X1 - 0.0599 (± 0.0592) X7 + 1.0876 (± 0.3812) X10 + 4.5352 (± 2.1729)						
		**x1**	**x7**	**x10**	**Observed**	**Predicted**
**S. No.**	**Compound**	**GATS6e**	**SpMax3_Bhp**	**IC2**	**PIC50**	**pIC50 (Y1)**
1	DenvInD_163	1.161267	3.785278	5.172004	6.754487	7.00296
2	DenvInD_226	0.999149	3.78578	4.929284	6.739929	7.148069
3	DenvInD_243	1.242125	3.59587	4.804338	5.692076	6.410378
4	DenvInD_219	0.977931	3.805944	5.169344	6.705534	7.461497
5	DenvInD_258	1.205206	3.778136	4.965562	6.785156	6.667976
6	DenvInD_298	0.885634	3.805317	5.132006	6.692504	7.653845
7	DenvInD_301	1.120817	3.785266	5.135271	7.167491	7.06509
8	DenvInD_250	1.250399	0	4.859946	6.436519	6.665371
9	DenvInD_232	1.245261	3.733813	4.790661	6.094204	6.379326
10	DenvInD_249	0.951123	3.803476	4.908748	6.742321	7.245872
11	DenvInD_266	0.831987	3.805306	5.038314	7.552842	7.687331
12	DenvInD_290	1.242125	3.59587	4.804338	5.013452	6.410378
13	DenvInD_299	1.16032	3.816199	5.068235	6.917215	6.890638
14	DenvInD_260	1.155745	3.778127	4.859946	6.468521	6.677931

15	DenvInD_229	1.221994	0	5.164541	6.829738	7.06833
16	DenvInD_225	1.272363	3.778121	4.759975	6.435334	6.274904
17	DenvInD_270	1.201916	3.798248	4.895607	6.730487	6.598993

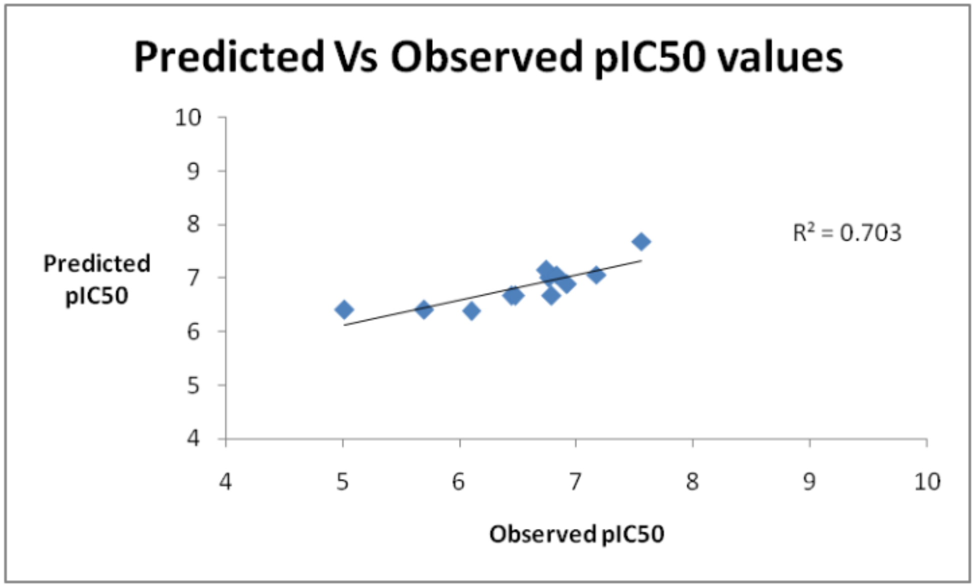

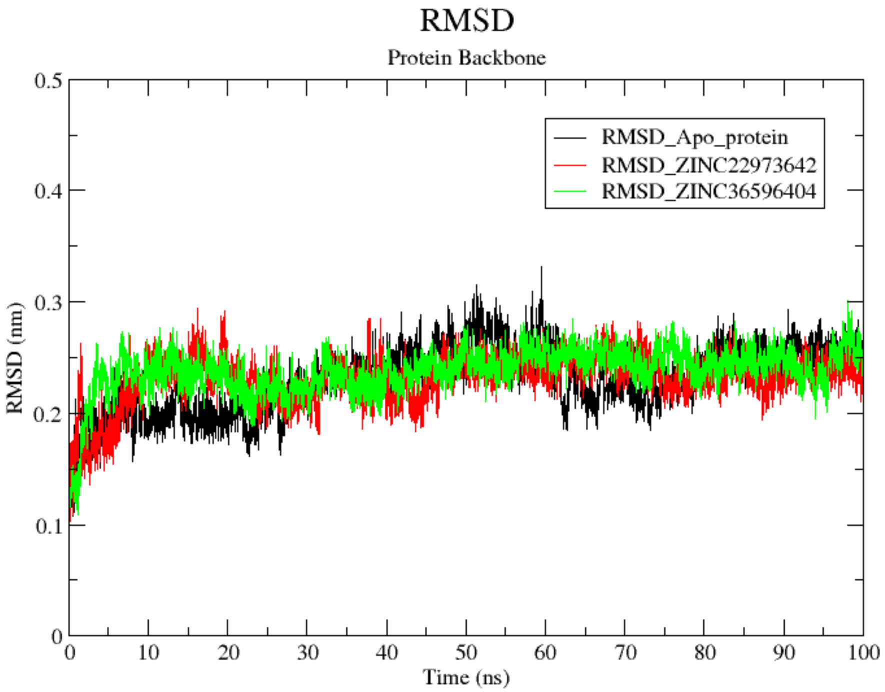

Figure 3:RMSD study plot of screened NS3 systems for 100 ns MD Simulation

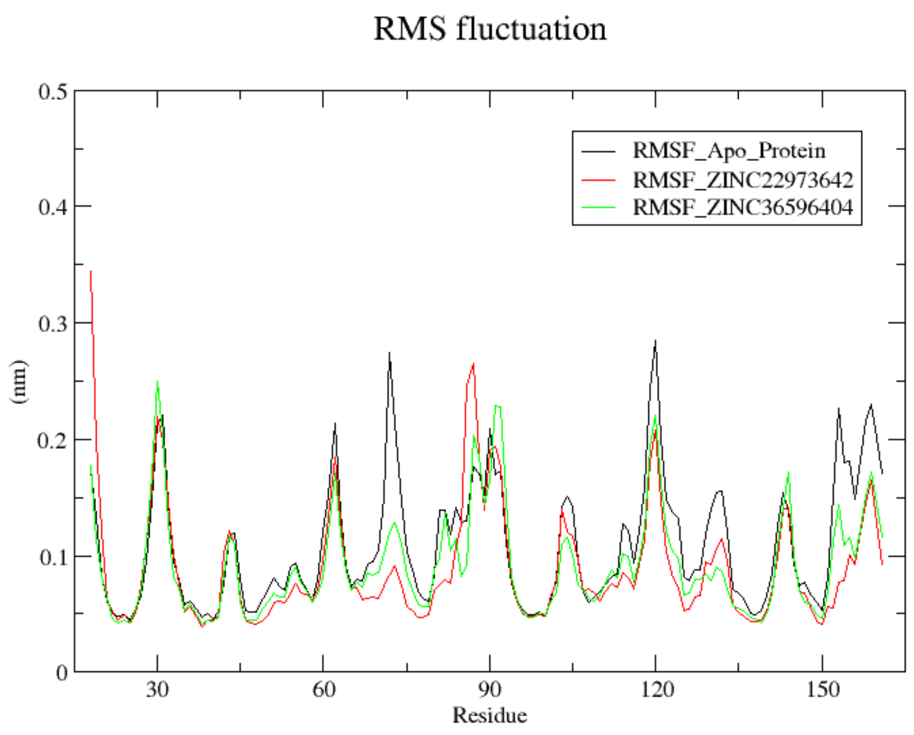

Figure 4: RMSF study plot of screened NS3 systems for 100 ns MD Simulation

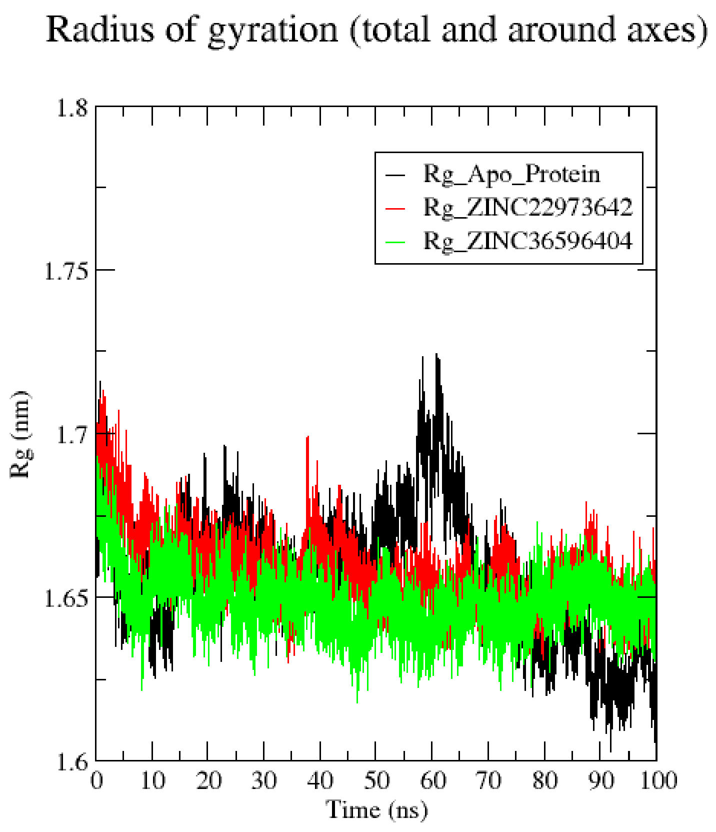

Figure 5: Radius of gyration of NS3 systems for 100ns

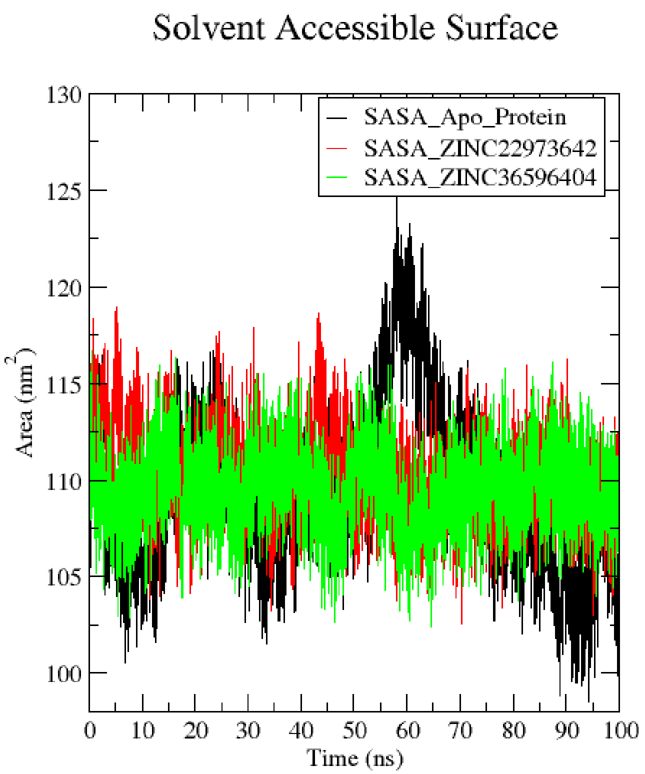

Figure 6: Solvent accessible surface area of NS3 systems

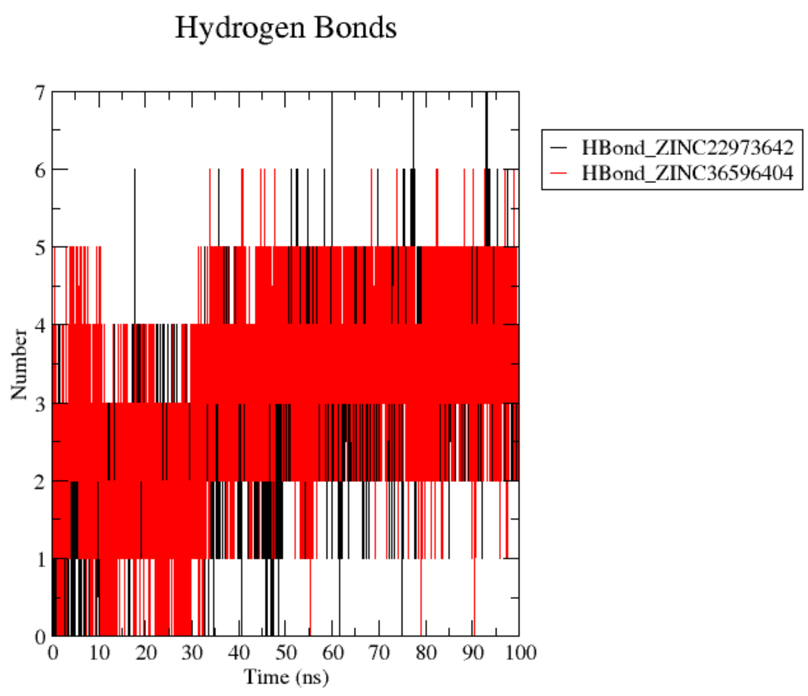

Figure 7: Hydrogen bond formation of NS3 systems for 100 ns simulation

Table showing amino acid interactions of standard drugs with dengue protease

S. No.	Compound	Amino Acids and Their Interactions
1.	Tipranavir	Van der waals: Ala164,Asn152,Ile165,Leu149,Gly148,Val147,Leu85<br>Pi-cation bond: Trp83,Trp69<br>Alkyl bond: Leu76
2.	Nelfinavir	Van der waals:Lys45,Leu11,Ile366<br>Carbon hydrogen bond: Thr35,Ile34,Lys61,Arg13<br>Attractive charges:Glu12,Leu53
3.	Asunaprevir	Conventional hydrogen bond: Leu115, Lys90<br>Van der waals: Gly124, Ala166<br>Carbon hydrogen bond: Trp89<br>Attractive charges: Glu91,Glu88,Asn167
4.	Darunavir	Van der waals: Trp69,Leu85,Val147,Glu88,Gly87,Gly148,Leu76,Ile165<br>Conventional hydrogen bond: Asn152<br>Pi-cation bond:Trp83
5.	Amprenavir	Van der waals: Gly87,Val147,Ala166,Glu88,Asn152,Leu76,Trp69<br>Conventional hydrogen bond:Trp83<br>Carbon hydrogen bond:Ile165<br>Pi-cation bond: Trp83

6.	Telaprevir	Van der waals:Asn167<br>Conventional hydrogen bond: Trp83,Asn152<br>Carbon hydrogen bond: Ala164,Ala166,Gly87,Trp69,Leu76,Val147,Ile165,Gly148<br>Attractive charges:Glu88
7.	Fosamprenavir	Van der waals:Gly148,Leu76,Val146,Val147,Ile165,Gly87,Ala166,Glu86<br>Conventional hydrogen bond: Leu149,Trp83,Leu85<br>Attractive charges: Glu88
8.	Lopinavir	Van der waals:Gly151,Asn152,Tyr150,Gly136,Ile77,Trp50,Leu76,Ser135<br>Conventional hydrogen bond: Thr48,Thr53,Val52<br>Carbon hydrogen bond:Asp75<br>Alkyl and pi-alkyl bond: Leu149,His51<br>Covalent bond: Trp50,Thr48
9.	Boceprevir	Van der waals: Ile34,Glu47,Glu49,Thr52<br>Conventional hydrogen bond: Thr35<br>Attractive charges: Glu12,Glu48,Leu53
10.	Ritonavir	Van der waals: Asn152,Ala164,Leu149,Ala166,Glu86,Gly87,Leu85,Asn167,Ala166,Leu76, Gly148,Val147,Trp83<br>Carbon hydrogen hydrogen bond: Ile165<br>Attractive charges: Glu88

11.	Atazanavir	Van der waals: Lys61,Thr35,Lys45,Arg13,Ala14,Ile36<br>Carbon hydrogen bond: Ile34<br>Attractive charges: Glu38,Leu53,Glu12
